# A multi-strategy improved tree–seed algorithm for numerical optimization and engineering optimization problems

**DOI:** 10.1038/s41598-023-37958-5

**Published:** 2023-07-04

**Authors:** Jingsen Liu, Yanlin Hou, Yu Li, Huan Zhou

**Affiliations:** 1 International Joint Laboratory of Intelligent Network Theory and Key Technology, Henan University, Kaifeng, China; 2 College of Software, Henan University, Kaifeng, China; 3 Institute of Management Science and Engineering, Henan University, Kaifeng, China; 4 Business School, Henan University, Kaifeng, China

**Keywords:** Engineering, Computational science

## Abstract

Tree–seed algorithm is a stochastic search algorithm with superior performance suitable for solving continuous optimization problems. However, it is also prone to fall into local optimum and slow in convergence. Therefore, this paper proposes an improved tree–seed algorithm based on pattern search, dimension permutation, and elimination update mechanism (PDSTSA). Firstly, a global optimization strategy based on pattern search is used to promote detection ability. Secondly, in order to maintain the diversity of the population, a random mutation strategy of individual dimension replacement is introduced. Finally, the elimination and update mechanism based on inferior trees is introduced in the middle and later stages of the iteration. Subsequently, PDSTSA is compared with seven representative algorithms on the IEEE CEC2015 test function for simulation experiments and convergence curve analysis. The experimental results indicate that PDSTSA has better optimization accuracy and convergence speed than other comparison algorithms. Then, the Wilcoxon rank sum test demonstrates that there is a significant difference between the optimization results of PDSTSA and each comparison algorithm. In addition, the results of eight algorithms for solving engineering constrained optimization problems further prove the feasibility, practicability, and superiority of PDSTSA.

## Introduction

Optimization problems are defined as finding the minimum or maximum value of a function consisting of many independent variables under certain constraints^1^. In recent years, the complexity and difficulty of optimization problems based on actual conditions have been increasing, and the traditional accurate solution methods are difficult to solve large-scale complex problems in a reasonable time. At the same time, heuristic intelligent optimization algorithms based on bionics are becoming more and more popular in solving complex problems due to their simple operation and high efficiency. In recent years, many heuristic algorithms inspired by nature/biology have been presented. For example, Kennedy et al.^2^ developed Particle Swarm Optimization (PSO) based on the foraging behavior of birds. Yang^3^ presented Bat Algorithm (BA) by simulating the echolocation of bats. Flower Pollination Algorithm put forward by Yang^4^ imitates the flower pollination process in nature. Inspired by the predatory behavior of humpback whales, Mirjalili et al.^5^ proposed Whale Optimization Algorithm (WOA). Inspired by the group behavior of salps, Mirjalili et al.^6^ also presented Salp Swarm Algorithm (SSA) and so on. These bionic algorithms provide new ideas for solving large-scale complex problems and are widely applied in many fields.

Tree–Seed Algorithm (TSA)^7^ is a new heuristic optimization algorithm proposed by Kiran in 2015. It is inspired by the relationship between trees and seeds, where trees spread their seeds to the surface during reproduction, and these seeds grow into new trees. The algorithm has the advantages of few adjustment parameters, easy implementation, and strong optimization ability. It has received extensive attention from scholars at home and abroad and has been successfully applied to power system forecasting^8^, land adjustment and redistribution^9^, power loss^10^, fuzzy cluster analysis^11^, clinical ultrasound images^12^, optimal power flow^13^, and other problems.

Experiments have shown that TSA can achieve ideal results in solving practical problems, but there is still room for further improvement in TSA’s ability to solve complex functions for global optimization. To this end, this paper presents an improved tree–seed algorithm based on pattern search, dimension permutation, and elimination update mechanism (PDSTSA). Firstly, a global optimization strategy based on pattern search is introduced, and the detection movement and pattern movement mechanisms are introduced into the global search stage, which effectively improves the detection ability and convergence speed of the algorithm. Secondly, a random mutation strategy of individual dimension permutation is introduced to enable individuals to mutate in a round of iterations to enhance the diversity of population positions. Finally, in the middle and late iterations, using the elimination update mechanism removes individuals with poor quality, and then generates fresh individuals with search potential to supplement the population, thereby enhancing the quality of the solution and improving the search performance of the algorithm. Eight representative algorithms are tested on 15 functions with different optimization characteristics in the CEC2015 suite for function extremum optimization in different dimensions. The optimization results indicate that the optimization accuracy and convergence speed of PDSTSA are significantly improved. The results of solving the two engineering optimization problems of alkylation unit optimal operation design and industrial refrigeration system design show that PDSTSA has good feasibility and applicability in dealing with engineering optimization problems.

The rest of this paper is organized as follows: “Related work” section provides relevant work, and “Basic tree–seed algorithm” section introduces a basic tree–seed algorithm. In “Improved strategies of PDSTSA” and “PDSTSA flow” sections respectively introduce the improved algorithm PDSTSA and algorithm process. In “Simulation experimental data analysis” section conducted simulation experiments and analyzed the solution results of PDSTSA on CEC2015 and engineering constrained optimization problems. Finally, in “Conclusion” section, the content of the paper and its future prospects are summarized.

## Related work

Optimization problems exist in many fields in the real world, and some problems require a lot of computation and have multiple limitations. Finding the optimal solution to these problems is challenging. And no algorithm can solve all problems, and the innovation and improvement of algorithms is a continuous process. Like other fundamental heuristic algorithms, TSA also has problems such as being prone to falling into local optima, unstable solution results, and imbalanced exploration and development capabilities. Therefore, many scholars have made corresponding improvements to address the shortcomings of TSA, with the aim of reducing the shortcomings of existing methods and improving the algorithm’s ability to solve different optimization problems.

Gharehchopogh et al.^14^ made a comprehensive review of TSA and its applications in different fields, covering many documents on the hybridization, improvement, mutation, and optimization of TSA.

Jiang et al.^15^ introduced hierarchical gravity learning and a stochastic-based migration mechanism to prevent the algorithm from getting into local optimal, enhance the population diversity, and better contribute to the balance between exploration and exploitation.

Beşkirli et al.^16^ introduced a tournament method to select the tree that generates seeds and optimized the search trend parameters to enable the algorithm to find more high-quality solutions in search space, improving the optimization accuracy and high-dimensional solution ability of the algorithm.

Babalik et al.^17^ used Deb’s rule to select the trees and seeds that survived in the next iteration, which had a good effect in minimizing the benchmark function and solving engineering constraint problems.

Jiang et al.^18^ presented an improved algorithm that dynamically determines the search trend and the number of seeds based on a feedback mechanism, which balances the algorithm’s global search and local search capabilities and improves the optimization efficiency.

Kiran et al.^19^ put forward four different approaches to improve tree and seed quality, significantly increasing optimization efficiency.

Ding et al.^20^ introduced the Lévy search mechanism to prevent the algorithm from falling into the local optimum and introduced the negative feedback mechanism into the seed update formula to further improve the search accuracy of high-dimensional optimization.

Horng et al.^21^ embedded ordinal optimization into TSA, which has good computational efficiency and quality in solving probability-constrained simulation optimization problems.

Cinar et al.^22^ improved TSA by using logic gates and similarity measurement techniques and solved the optimization problem with continuous structure solution space, showing good solution quality and robustness of the algorithm.

Gungor et al.^23^ used five update rules and one wilting process to obtain trees and seeds, which enhanced the global search and local search ability of the algorithm and performed well in solving high-dimensional continuous problems.

## Basic tree–seed algorithm

In basic TSA, the specific algorithm flow is as follows:

**Step 1:** Initialize the location of each tree in the population. Use Eq. (1) to generate a feasible solution as the location of the initial tree $T_{i,j} (i = 1,2 \ldots {\text{N}},\;j = 1,2 \ldots {\text{D}})$.

1 $$T_{i,\,j} = L_{j,\,\min } + r_{i,\,j} \times (H_{j,\,\max } - L_{j,\,\min } )$$

where ${\text{N}}$ is the population size of the tree, ${\text{D}}$ is the spatial dimension, and $T_{i,j}$ refers to the location of the $j{\text{th}}$ dimension for the $i{\text{th}}$ tree. $L_{j,\min }$ and $H_{j,\max }$ are the lower and upper bounds of the $j{\text{th}}$ dimension, respectively, and $r$ indicates an ${\text{N}} \times {\text{D}}$ matrix composed of uniformly distributed random numbers between $(0,1)$.

**Step 2:** Enter the iteration, compute the fitness value of each tree according to the objective function, and then select the tree with the best location.

**Step 3:** Determine the number of seeds each tree produces according to Eq. (2).

2 $$ns = fix(low + (high - low) \times rand) + 1$$

where $fix$ is a function that rounds an element to the nearest integer toward zero, $low$ refers to the minimum number of seeds produced from a tree, and its value is ${\text{N}} \times 10\%$, $high$ refers to the maximum number of seeds produced from a tree, and its value is ${\text{N}} \times 25\%$.

**Step 4:** For each tree, the location of each seed is generated through Eqs. (4) and (5).

3 $$\alpha_{i,j} = (rnd - 0.5) \times 2$$

$$S_{i,j} = \left\{ {\begin{array}{*{20}l} {T_{i,j} + \alpha_{i,j} \times (B_{j} - T_{r,j} )} \hfill & {rand < \, ST} \hfill & \qquad {(4)} \hfill \\ {T_{i,j} + \alpha_{i,j} \times (T_{i,j} - T_{r,j} )} \hfill & {rand \ge \, ST} \hfill & \qquad {(5)} \hfill \\ \end{array} } \right.$$

In Eq. (3), $rnd$ represents a random number in the range of (0, 1), so $\alpha_{i,j}$ represents the scaling factor randomly generated in the range of (− 1, 1). The value of ${\text{ST}}$ in Eqs. (4) and (5) are 0.1, $S_{i,j}$ represents the $j{\text{th}}$ dimension of the current seed generated for the $i{\text{th}}$ tree, $B_{j}$ is the $j{\text{th}}$ dimension of the best tree location, and $T_{r,j}$ refers to the $j{\text{th}}$ dimension of the $r{\text{th}}$ tree randomly selected from the tree population, where $i$ and $r$ are different indices.

**Step 5:** Compute the fitness value of all seeds generated by the tree through objective function, select the optimal seed, and compare it with the current tree. If the seed is better than the tree, this seed will replace the tree.

**Step 6:** Judge whether the termination conditions are met. If yes, output the final result; Otherwise, go to step 2 for the next iteration.

## Improved strategies of PDSTSA

### Global optimization mechanism based on pattern search

In TSA, the global search is a random search based on the current position and any random individual. During the optimization process, the movement of the individual has no directionality, resulting in a slow overall convergence speed. The pattern search method^24^ is a direct search method that does not rely on derivatives, and its two critical steps are detection movement and pattern movement. Detection movement is changing the value on a single dimension of an individual to move it in a positive or negative direction for a specified step size. The pattern movement is to accelerate the search along the direction of fitness value decline. The pattern search method is relatively flexible in its search process, and the search trend is directional. Inspired by the pattern search method, this paper proposes a global optimization strategy based on pattern search in PDSTSA, which introduces the detection movement and pattern movement mechanism into the global search stage, effectively improving the detection ability and convergence speed of the algorithm.

For the detection movement process, it is required that in the global search stage, the position formula of seeds generated by a tree is divided into positive direction search and negative direction search based on step size. For the pattern moving process, initialize the seeds of all trees to search in the positive direction of step size. If a new seed replaces the tree after one iteration, the tree is still searching in the positive direction in the next iteration. If the current tree does not produce a better seed, the tree will search in the negative direction of the step size in the next iteration and reduce the search step size by a certain proportion. In this way, the algorithm can quickly determine the direction in which the fitness value decreases and effectively improve the convergence speed of the algorithm. In the iterative process, the search step size is gradually reduced, thereby increasing the local search ability in the later stage and improving the optimization accuracy of the algorithm.

Set a judgment flag $jud$ to determine whether a seed replaced a tree in the previous iteration. Initialize the $jud$ of all trees to 1. If a better seed replaces the tree after one iteration, let $jud = 0$. If the tree has not been replaced, let $jud = 0$. According to the value of $jud$, determine the direction along which the formula searches for the position of the seed generated by the next iteration of the current tree. The search position of the seed in the positive and negative directions is shown in Eqs. (6) and (7).

6 $$S_{i,j} = T_{i,j} + rand \times S \times (T_{i,j} - T_{r,j} )$$

7 $$S_{i,j} = T_{i,j} - rand \times S \times (T_{i,j} - T_{r,j} )$$

8 $$S = S \times k$$

In Eqs. (6) and (7), $T_{i,j}$ denotes the $j{\text{th}}$ dimension position value of the $i{\text{th}}$ tree, $rand$ is a random number uniformly distributed in the (0, 1) interval. $S$ represents the control factor of the search step length, and its initial value is 2, which decreases in a certain proportion during the optimization process. where $k$ is a constant less than 1, and the value in this paper is 0.97. If the tree doesn’t produce better seeds, $S$ decreases by a certain proportion according to Eq. (8). By reducing the step length, the pattern search process is more precise, and the moving direction of the population is closer to the optimal direction. At the same time, the seeds are deeply searched in the small neighborhood of the tree in the later period of iteration, which effectively improves the optimization accuracy of the algorithm.

### Random mutation mechanism of individual dimension

In order to maintain the overall search capability of the population, this paper introduces a random mutation strategy of individual dimension permutation^25^ in PDSTSA, which enables individuals to mutate during the process of one round of iteration to increase the diversity of population positions and enhance the ability of individuals to jump out of local minima. In PDSTSA, the position obtained by comparing and replacing the $ith$ tree with the seed is set as $T_{i}$, and its fitness value is $obj\_T_{i}$. Two of the ${\text{D}}$ dimensions of this tree are randomly selected for exchange. Repeat this operation for $m$ times to obtain the mutated position $T_{i} \_mutation$. Calculate the fitness value $obj\_T_{i} \_mutation$ after mutation, and if $obj\_T_{i} \_mutation < obj\_T_{i}$, set $T_{i} = T_{i} \_mutation$. In this way, the optimal individuals are retained after the mutation is completed, and the population quality is raised. While increasing the diversity of the population, it can also improve the convergence speed, in which the number of permutation ${\text{m}}$ is a random integer on^1,4^.

### Elimination and update mechanism based on poor location

In the biological evolution process of nature, competitive individuals can survive, and uncompetitive individuals will be eliminated. The evolutionary algorithm population is also divided into competitive individuals and uncompetitive individuals. This paper introduces an elimination update mechanism based on poor position in the PDSTSA. If the fitness value obtained by an individual is poor compared to the majority of the population, it indicates that the adaptability in competition is poor and there is no ability to search for the optimal position. Therefore, those individuals with poor quality are eliminated. Then fresh individuals with search potential are generated to supplement the population, thereby enhancing the quality of the solution and improving the search performance of the algorithm. The specific operation strategy is as follows:

After the tree $T_{i}$ passes the individual dimension mutation strategy, if the current iteration number is more than half of the total iteration number, then the update strategy for $T_{i}$ is executed. The population is sorted in ascending order according to the fitness value. The individuals in the front ${2 \mathord{\left/ {\vphantom {2 3}} \right. \kern-0pt} 3}$ are set as the dominant group, and the rest $1/3$ individuals are the inferior groups. Judge whether the current tree is a disadvantaged group. If so, the tree will be eliminated, and then a new individual will be generated according to Eq. (9) to replace the eliminated tree and add it to the population.

9 $$T_{i} = T_{k} + (T_{i} - T_{k} ) \times \alpha$$

where $T_{i}$ represents the position of the current tree, and $T_{k}$ represents the position of the tree randomly selected from the dominant group. $\alpha$ is the random parameter matrix calculated according to Eq. (3).

At the later stage of the iteration, the individuals in the population have evolved to a better position, so although $T_{k}$ is only a random position in the dominant population, the position near $T_{k}$ still has better competitiveness and search potential. After the elimination and update mechanism of inferior trees, the overall quality of the population has been improved, further improving the search efficiency and convergence accuracy of the algorithm in the later stage of iteration.

## PDSTSA flow

PDSTSA algorithm is described as follows:
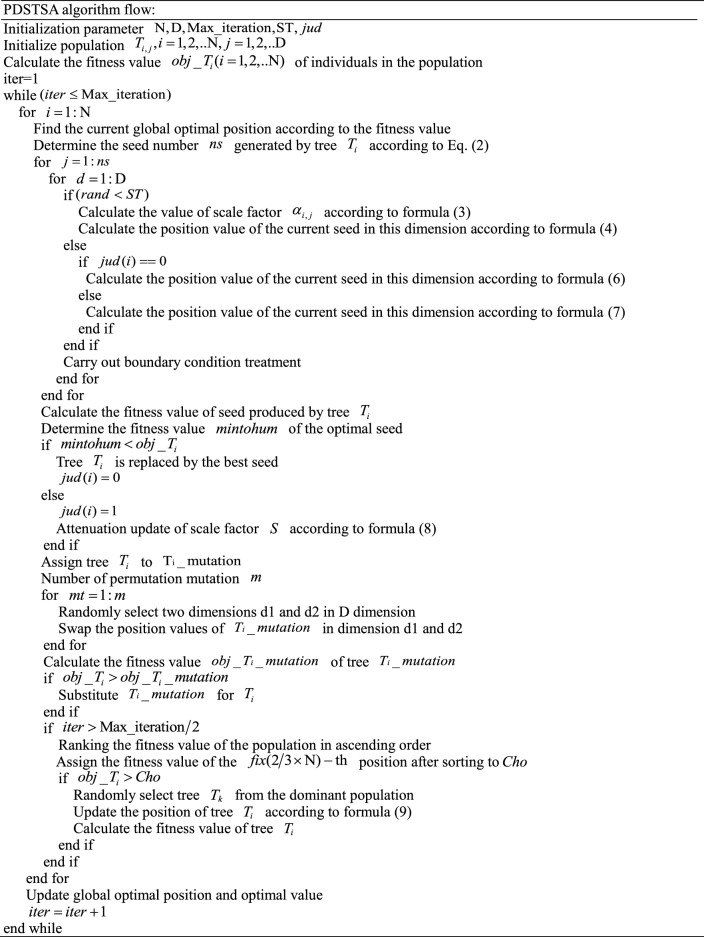


## Simulation experimental data analysis

In this section, the extensive and authoritative IEEE CEC2015^26^ test function suite is used to evaluate the performance of the PDSTSA algorithm proposed in this paper, and PDSTSA is compared with the basic tree–seed algorithm (TSA, 2015)^7^, the improved TSA based on intelligent search mechanisms (NTSA, 2021)^19^, the enhanced TSA via feedback mechanism (fbtsa, 2020)^18^, the whale algorithm (WOA, 2016)^5^, the elite reverse gold sinusoid whale algorithm (EGSWOA, 2019)^27^, the grey wolf algorithm (GWO, 2014)^28^, and variant differential evolutiona (JADE, 2009)^29^, and the comparative tests are carried out in 30, 50 and 100 dimensions, respectively.

### Test function

The specific test functions are all 15 functions in the CEC2015 suite. Table 1 shows the main features of these functions. $f(x^{{_{ * } }} )$ represents the theoretical optimal value of the function, the theoretical optimal values of functions F1–F15 are 100–1500, and the value range of independent variables of all test functions is [− 100, 100]. These functions can be divided into four categories according to different characteristics, of which F1–F2 are unimodal functions, F3–F5 are multimodal functions, F6–F8 are hybrid functions, and F9–F15 are composition functions. These 15 functions are all shifted and rotated based on the basic functions, so they are difficult to solve and suitable for testing the optimization performance of the algorithm.

**Table 1:** Characteristics of CEC2015 benchmark test functions.

Function	Category	Description	f(x*)
F1	Unimodal	Rotated high conditioned elliptic function	100
F2	Unimodal	Rotated cigar function	200
F3	multimodal	Shifted and rotated Ackley’s function	300
F4	multimodal	Shifted and rotated Rastrigin’s function	400
F5	multimodal	Shifted and rotated Schwefel’s function	500
F6	Hybrid	Hybrid function 1 (N = 3)	600
F7	Hybrid	Hybrid function 2 (N = 4)	700
F8	Hybrid	Hybrid function 3 (N = 5)	800
F9	Composition	Composition function 1 (N = 3)	900
F10	Composition	Composition function 2 (N = 3)	1000
F11	Composition	Composition function 3 (N = 5)	1100
F12	Composition	Composition function 4 (N = 5)	1200
F13	Composition	Composition function 5 (N = 5)	1300
F14	Composition	Composition function 6 (N = 7)	1400
F15	Composition	Composition function 7 (N = 10)	1500

### Optimization accuracy analysis

In order to test the optimization ability of the PDSTSA, this paper sets the dimensions of test functions F1–F15 as D = 30/50/100, respectively. In order to ensure the fairness and objectivity of the experiment, the eight algorithms are independently run 30 times under the same conditions, the population size is 100, and the maximum iteration times is 1000. The experimental test platform is Windows 10 operating system, and the programming language is Matlab R2018a. In terms of algorithm parameter setting, no additional parameters need to be set for the eight algorithms, which is consistent with the original literature and algorithm source code. Table 2 shows the average, optimal, and worst values of the optimization results obtained by each algorithm running 30 times under the conditions of 30, 50, and 100 dimensions. Among them, the best results of each data are marked in bold.

**Table 2:** Comparison of optimization results of 6 algorithms under fixed iteration times.

Function	Algorithm	D = 30			D = 50			D = 100		
		Average value	Optimal value	Worst value	Average value	Optimal value	Worst value	Average value	Optimal value	Worst value
F1	PDSTSA	**58216**.**08**	**15543**	**188731**.**51**	**896246**.**36**	**539680**.**96**	**1515462**.**39**	**5670802**.**01**	**3207991**.**72**	**8,953,675**
	TSA	26395209.39	14802076.67	38654639.87	395858732.7	190463498.6	504928330.7	2,562,696,016	1,420,405,027	3,001,814,529
	fbtsa	601339.36	215492.75	1170757.8	4557226.1	1461958.01	9786952.49	30452598.74	17711542.74	52405045.74
	NTSA	4010615.4	2062886.05	6479987.7	21966209.17	14612095.46	34147759.8	122780881.1	96076249.85	157067224.6
	WOA	77071178.32	17354805.4	174875374.7	126133030.1	31980749.23	293730461.2	565452388.2	322808939.9	786,150,578
	EGSWOA	26969651.12	4629502.97	60750323.66	60653746.24	25024565.18	110567070.4	239950601.8	128804760.7	391225489.3
	GWO	25019035.45	6048220.57	63700047.22	74136572.53	26730937.73	175177145.20	301241946.7	155558815.2	476673003.5
	JADE	439178878.97	53045.35	1133772943.95	994375095.6	1411733.541	7,649,865,410	2,024,707,532	7443740.378	16,765,368,826
F2	PDSTSA	**200**.**11**	**200**	**202**.**94**	**200**.**67**	**200**	**204**.**2**	**960**.**34**	204.89	**3947**.**77**
	TSA	1378.32	200.58	5750.96	7921.29	236.96	28883.04	6,329,876,258	4,874,999,516	8,241,023,106
	fbtsa	1058.54	389.63	2256.3	4117.13	1003.21	8290.71	29380.91	6299.97	57408.45
	NTSA	449.51	201.18	1173.83	2176.75	305.95	7570.01	2675.72	754.92	8208.56
	WOA	103571950.3	26318771.55	228552302.6	1,184,348,898	509138529.1	2,578,107,801	20,040,837,120	12,458,447,245	32,833,765,801
	EGSWOA	10888539.05	614513.19	27707297.95	249837769.5	49031793.95	484968944.9	5,152,283,063	2,310,645,356	9,050,879,527
	GWO	1646131366.27	98647214.14	6059860274.29	5,558,633,879	2,399,205,026	10,680,412,187	31,612,072,714	15,026,524,626	60,331,565,418
	JADE	7931722077.76	200.00	75259226440.68	8,716,011,116	200.0357708	2.6148E + 11	2637.584944	**201**.**9436359**	8537.223132
F3	PDSTSA	**320**.**09**	**320**.**05**	**320**.**12**	**320**.**35**	**320**.**24**	**320**.**43**	320.89	320.78	320.96
	TSA	320.85	320.77	320.94	321.09	320.98	321.15	321.31	321.25	321.34
	fbtsa	320.9	320.82	320.97	321.12	321.07	321.18	321.32	321.25	321.36
	NTSA	320.28	320.23	320.33	320.48	320.4	320.52	**320**.**79**	320.75	**320**.**83**
	WOA	320.56	320.27	320.86	320.8	320.6	321.06	321.07	320.85	321.22
	EGSWOA	320.32	320.11	320.6	320.67	320.44	320.91	320.95	**320**.**71**	321.08
	GWO	320.9805486	320.79513	321.0724896	321.1842538	321.0917953	321.2471833	321.3605277	321.3071267	321.3984082
	JADE	320.9044734	320.6996816	321.1741686	321.0706527	320.7373352	321.3488263	321.363745	321.1031477	321.4932727
F4	PDSTSA	462	436.81	481.59	545.42	465.67	580.09	**565**.**53**	**518**.**4**	**636**.**83**
	TSA	580.65	567.36	597.35	804.37	777.22	825.7	1474.28	1406.47	1503.81
	fbtsa	**425**	**413**.**93**	**437**.**81**	**461**.**66**	**438**.**8**	**511**.**17**	632.97	557.06	814.03
	NTSA	460.12	447.98	472.79	571.06	539.35	607.83	1074.97	1006.33	1143.45
	WOA	686.04	564.71	836.67	950.18	820.71	1109.06	1654.49	1461.35	1873.59
	EGSWOA	619.18	547.95	706.2	817.71	718.78	963.16	1440.82	1286.41	1595.6
	GWO	494.1407261	431.7818202	627.4932582	603.5996821	533.1447128	657.9184719	997.4437451	865.7661445	1109.756173
	JADE	613.951026	528.1273331	762.6872919	795.0632897	675.6532701	1522.776564	1299.401789	1152.853885	1546.953029
F5	PDSTSA	**2196**.**52**	**1375**.**61**	**2699**.**11**	**4360**.**83**	**2738**.**64**	**5080**.**82**	**13385**.**29**	**10324**.**48**	19475.45
	TSA	7030.48	6578.86	7453.16	13396.92	12365.24	14045.23	30469.49	29366.57	31148.86
	fbtsa	5896.26	3179.31	6779.45	11999.87	3290.15	13115.28	29929.96	27037.32	30874.74
	NTSA	2693.02	2008.2	3095.24	5510.3	4542.77	6152.89	16036.85	14749.94	**17296**.**06**
	WOA	5280.97	4058.63	6981.81	10306.29	8162.5	13482.11	24545.35	20353.59	29163.65
	EGSWOA	4639.24	3124.85	5932.98	8737.06	6723.87	11186.23	21663.76	16158.54	27550.22
	GWO	3514.488434	1818.89912	8444.293049	6512.196626	4508.644564	14200.45878	16611.66392	12632.60188	31773.00538
	JADE	7834.85169	6415.887728	9278.807683	14451.22903	11840.91244	16594.57015	32675.43333	27691.05017	34938.46684
F6	PDSTSA	**9251**.**78**	**1328**.**17**	**34835**.**64**	**60124**.**1**	**12288**.**46**	**130139**.**56**	**1279395**.**27**	**744117**.**68**	**2421464**.**39**
	TSA	1287480.79	690361.53	2548890.24	16431047.05	7986607.44	22672305.33	202437707.2	110378896.5	262482056.1
	fbtsa	73390.95	16814.13	214813.94	523807.2	210816.55	1112242.6	5854132.68	2746311.52	11021103.08
	NTSA	1015895.25	347447.49	1909829.17	3657659.87	1385331.91	6206507.68	25884674.36	18058050.38	33827878.53
	WOA	2811663.89	555365.75	7400326.05	24219458.24	8370844.42	54320238.97	77446467.74	33491333.14	162510827.5
	EGSWOA	1112514.73	63150.21	3434439.75	6022727.44	2324741.25	16549153.94	25826173.51	12880119.78	51033039.32
	GWO	1798013.627	34293.9668	9707577.335	4908117.611	567745.5197	14387284.24	19183689.35	7322352.578	46800479.34
	JADE	45808366.68	15246386.32	92622741.62	225314710.9	58059.00298	620731239.6	848655856.3	1150401.579	2786798044
F7	PDSTSA	**703**.**92**	**702**.**58**	**705**.**59**	**709**.**11**	**706**.**03**	**713**.**01**	**776**.**32**	**726**.**02**	**810**.**99**
	TSA	712.38	710.77	713.34	757.82	755.21	759.22	870.59	851.76	873.45
	fbtsa	707.34	703.77	709.47	741.79	706.96	776.57	847.27	805.49	863.03
	NTSA	708.52	705.98	710.81	720.64	714.39	728.69	809.4	778.12	836.02
	WOA	744.56	719.73	800.41	817.53	756.86	955.12	1240.76	1030.5	1472.72
	EGSWOA	726.96	714.45	790.97	793.98	736.38	840.45	1063.49	899.48	1249.8
	GWO	719.9140362	714.6793785	727.3792889	798.1201511	758.4370087	899.8418302	1063.463004	934.0420271	1206.76647
	JADE	777.6586256	711.0506827	1427.247785	1107.012978	741.9848344	2963.854717	1095.852177	812.3027242	3499.004183
F8	PDSTSA	**1704**.**33**	**868**.**93**	**5183**.**93**	**53241**.**26**	8524.88	**204264**.**13**	**320103**.**69**	**110531**.**66**	**620574**.**73**
	TSA	178482.36	58713.06	318465.83	5023363.19	1640093.58	7971822.42	84294173.21	47178433.97	110713261.4
	fbtsa	23852.95	6285.88	44088.44	377020.97	142080.15	721588.62	3649561.8	1382089.12	5767195.49
	NTSA	170142.64	37230.16	403559.95	2433732.48	665769.61	5247056.75	15188918.35	8452096.28	23300617.1
	WOA	647627.73	66870.53	1545314.31	9391306.38	2302363.36	16391929.86	29141091.26	11580882.29	56964357.84
	EGSWOA	129668.53	16079.36	540238.86	3650358.45	1012770.99	9582307.58	13487593.74	6777513.18	21840383.31
	GWO	362272.5289	36489.3448	1129435.825	2669264.262	882373.4177	17126601.08	16969141.46	6008868.52	31379717.75
	JADE	15965188.82	807663.8687	31944228.97	87702477.35	**3637**.**505584**	198774619.7	491740160.7	450341.7281	1,246,278,007
F9	PDSTSA	1002.68	1002.42	1002.95	**1004**.**42**	**1003**.**99**	**1004**.**97**	**1008**.**3**	**1007**.**87**	**1008**.**87**
	TSA	1003.19	1002.94	1003.41	1006.31	1005.84	1006.95	1017.78	1015.38	1020.15
	fbtsa	**1002**.**5**	**1002**.**14**	**1002**.**87**	1004.94	1004.41	1005.46	1011.39	1009.19	1012.96
	NTSA	1003.63	1003.33	1003.91	1006.82	1006.23	1007.31	1013.21	1012.39	1013.95
	WOA	1039.96	1005.59	1513.42	1039.88	1011.01	1738.91	1366.48	1061.88	3351.93
	EGSWOA	1006.21	1004.28	1007.48	1038.33	1009.53	1807.06	1117.95	1018.23	2387.96
	GWO	1012.337154	1003.278941	1185.006172	1023.915149	1008.088044	1047.191593	1105.617874	1079.645158	1142.05487
	JADE	1110.737291	1003.862076	1272.833292	1314.786844	1006.637943	1765.174252	1691.532079	1012.364647	3101.704739
F10	PDSTSA	**5615**.**37**	2091.3	**20315**.**89**	**6388**.**15**	**2335**.**2**	**22169**.**06**	**27717**.**07**	19421.55	**44034**.**01**
	TSA	483035.89	206434.54	996767.65	1035451.07	519171.55	1846766.31	1391222.95	449560.71	3015990.39
	fbtsa	63973.91	26540.09	143499.55	48434.53	10843.51	130612.05	82656.69	19356.27	190575.39
	NTSA	320342.37	125011.79	549550.23	807260.91	258576.3	1607992.47	3137742.25	1345180.1	5128137.8
	WOA	5012138.16	699725.44	12548188.98	9763345.17	1291742.94	20865371.58	42072322.3	7814266.72	86192037.19
	EGSWOA	1743868.11	248799.23	3923640.05	4315927.87	1116597.61	10257974.63	16827534.99	4373192.86	39405852.96
	GWO	2162621.365	234702.3724	3872252.072	5932740.164	571454.4085	12302347.8	38910488.12	5522552.672	82908804.58
	JADE	38361815.03	**1866**.**824621**	89672659.61	111828790.9	3969.596512	313852341.1	681600574.3	**12117**.**27332**	2,630,382,285
F11	PDSTSA	**1401**.**25**	**1400**.**87**	**1402**.**09**	**1403**.**82**	**1401**.**46**	**1409**.**32**	**1423**.**28**	**1413**.**62**	**1442**.**11**
	TSA	1613.69	1466.2	1759.76	2722.34	2570.99	2842.19	5006.11	4855.03	5130.83
	fbtsa	1438.34	1402.79	1500	1521.86	1500.01	1593.93	2520.7	2115.81	2942.43
	NTSA	1430.42	1412.16	1452.37	1611.91	1445.37	2316.43	2388.66	1580.97	3831.92
	WOA	2296.09	1486.54	2738.74	3366.97	3147.74	3564.74	5779.58	5335.19	6347.56
	EGSWOA	1765.85	1425.65	2536.27	2773.48	1449.9	3518.37	5588.44	5029.6	5933.61
	GWO	1855.480988	1732.479228	1992.979684	2208.164039	2030.062651	2369.12002	3681.356453	3345.777154	4011.452249
	JADE	2519.401497	2390.009586	2746.175019	3281.887435	3048.253298	3571.748679	5111.033272	2614.722549	5876.565736
F12	PDSTSA	**1304**.**7**	1303.89	**1305**.**29**	1310.68	**1306**.**3**	1400.01	1322.31	**1312**	1400.02
	TSA	1306.67	1305.66	1307.52	1315.31	1311.23	1365.44	1358.66	1324.57	1400.41
	fbtsa	1304.72	**1303**.**68**	1305.39	**1307**.**98**	1306.44	**1309**.**08**	**1318**.**4**	1316.3	**1320**.**29**
	NTSA	1306.24	1305.4	1306.98	1310.12	1308.37	1314.1	1376.16	1317.38	1400.02
	WOA	1320.21	1308.09	1400.37	1394.59	1314.62	1400.27	1395.57	1326.74	1400.57
	EGSWOA	1313.61	1305.92	1400.37	1391.65	1309.45	1400.29	1400.5	1400.42	1400.67
	GWO	1309.622228	1305.040974	1400.497392	1373.776943	1308.943991	1400.726916	1374.067025	1323.326037	1403.858853
	JADE	1388.748443	1307.71071	1401.124434	1400.284956	1400.270239	1400.55231	1400.502653	1400.406159	1401.793268
F13	PDSTSA	1300.01	1300.01	1300.01	1300.01	1300.01	1300.01	1300.02	1300.02	1300.02
	TSA	1300.03	1300.03	1300.03	1300.07	1300.07	1300.08	1300.07	1300.07	1300.07
	fbtsa	1300.03	1300.03	1300.03	1300.1	1300.09	1300.11	1300.11	1300.09	1300.15
	NTSA	**1300**.**01**	**1300**.**01**	**1300**.**01**	**1300**.**01**	**1300**.**01**	**1300**.**01**	**1300**.**02**	**1300**.**02**	**1300**.**02**
	WOA	1300.04	1300.03	1300.07	1300.29	1300.11	1300.66	1300.25	1300.08	1300.47
	EGSWOA	1300.04	1300.03	1300.1	1300.23	1300.1	1300.54	1300.19	1300.08	1300.54
	GWO	1300.042025	1300.02951	1300.066559	1300.286894	1300.175682	1300.55833	1300.927154	1300.313438	1302.626173
	JADE	1300.041075	1300.03101	1300.178798	1300.834561	1300.107503	1311.129251	1300.098751	1300.077329	1300.684348
F14	PDSTSA	**9742**.**42**	6153.38	**18283**.**51**	**51841**.**07**	49119.62	59459.63	**100479**.**08**	96439.2	**103809**.**14**
	TSA	34675.57	34385.24	34978.39	61274.2	60423.99	72201.51	110589.3	110506.15	110672.64
	fbtsa	33323.95	32782.58	34045.77	56946.91	50915.7	74961.28	115761.27	113355.84	123467.18
	NTSA	22440.61	**5177**.**16**	32775.33	52618.41	**16426**.**91**	**58643**.**08**	102754.37	**95772**.**05**	105617.07
	WOA	38960.37	33562.11	45439.87	85343.64	61556.69	95411.25	170036.28	138360.22	205707.38
	EGSWOA	37574.43	33193.62	39865.98	76988.5	53918.68	86471.46	155546.09	119038.77	191827.5
	GWO	36125.76048	33369.6873	41686.91166	78758.34275	68678.05485	84308.55622	180670.1207	165547.2245	196114.9872
	JADE	46260.22257	37408.67886	61633.83382	93688.18343	86150.14944	128635.8898	180035.6879	154911.4151	277265.616
F15	PDSTSA	**1600**	**1600**	**1600**	**1600**	**1600**	**1600**	1603.47	1602.4	1604.71
	TSA	**1600**	**1600**	**1600**	**1600**	**1600**	**1600**	2681.01	2059.91	3222.24
	fbtsa	**1600**	**1600**	**1600**	**1600**	**1600**	**1600**	**1601**.**14**	**1600**.**37**	**1602**.**71**
	NTSA	**1600**	**1600**	**1600**	**1600**	**1600**	**1600**	1606.6	1605.99	1607.41
	WOA	1625.92	1612.44	1670	1656.95	1624.84	1810.87	3732.07	2443.84	6331.86
	EGSWOA	1611.08	1604.3	1629.27	1623.01	1617.31	1642.06	1856.78	1681.38	2163.99
	GWO	1623.046456	1608.467868	1690.354504	1694.888516	1618.467224	2077.522572	3467.803017	1855.367066	7841.636459
	JADE	9628.564226	1600	188106.6705	97344.87405	1600	1266336.466	210401.259	1601.05472	3479001.909

From Table 2, it can be seen that on the unimodal test functions F1 and F2, the average, optimal, and worst values of PDSTSA are significantly better than other comparison algorithms in different dimensions for F1. For F2, when D = 30/50, the optimal value of PDSTSA can converge to the theoretical optimal value, and when D = 100, the average and worst values of PDSTSA are better than the comparison algorithm, and the optimal value is only second to JADE, demonstrating superior global exploration ability. On the multimodal test functions F3–F5, for F3 and F5, the average value, the optimal value, and the worst value obtained by PDSTSA under the condition of D = 30/50 are the best of the eight algorithms. For F4, the optimization results obtained by PDSTSA under the condition of D = 100 are also better than the comparison algorithm. For the other dimensions of these three functions, PDSTSA is one of the eight algorithms with better results, which is superior to most comparison algorithms. On the mixed functions F6–F8, for F6 and F7, PDSTSA outperforms the comparison algorithm in terms of average, optimal, and worst values under different dimensional conditions, demonstrating superior optimization performance and dimensional adaptability. For F8, PDSTSA is the best among the eight algorithms under the condition of D = 30/100, and under the condition of D = 50, the optimal value of PDSTSA is second only to JADE, and the average and worst values are better than other algorithms. On the composition functions F9–F15, for F10 and F11, PDSTSA’s solution results under the condition of D = 30/50/100 are the best among the eight algorithms, with high optimization accuracy and stability. For F9, F12, and F14, the average, the best or worst value of multiple optimization results of PDSTSA under different dimensions is the best of the eight algorithms, and the overall performance is better. For F13 and F15, the optimization results of the eight algorithms are similar, and the solving ability is equivalent. From the experimental results of each algorithm solving all 15 test functions in the CEC2015 suite, it can be seen that PDSTSA shows a relatively superior optimization effect as a whole.

From the above analysis, it is clear that PDSTSA has sufficient exploration and exploitation capabilities to solve unimodal, multimodal, hybrid, and combinatorial problems. Compared with other algorithms, its optimization accuracy, solution stability, and adaptability to different functions and dimensions have obvious advantages, and PDSTSA has obtained more competitive results.

### Convergence curve analysis

**Figure 1:**
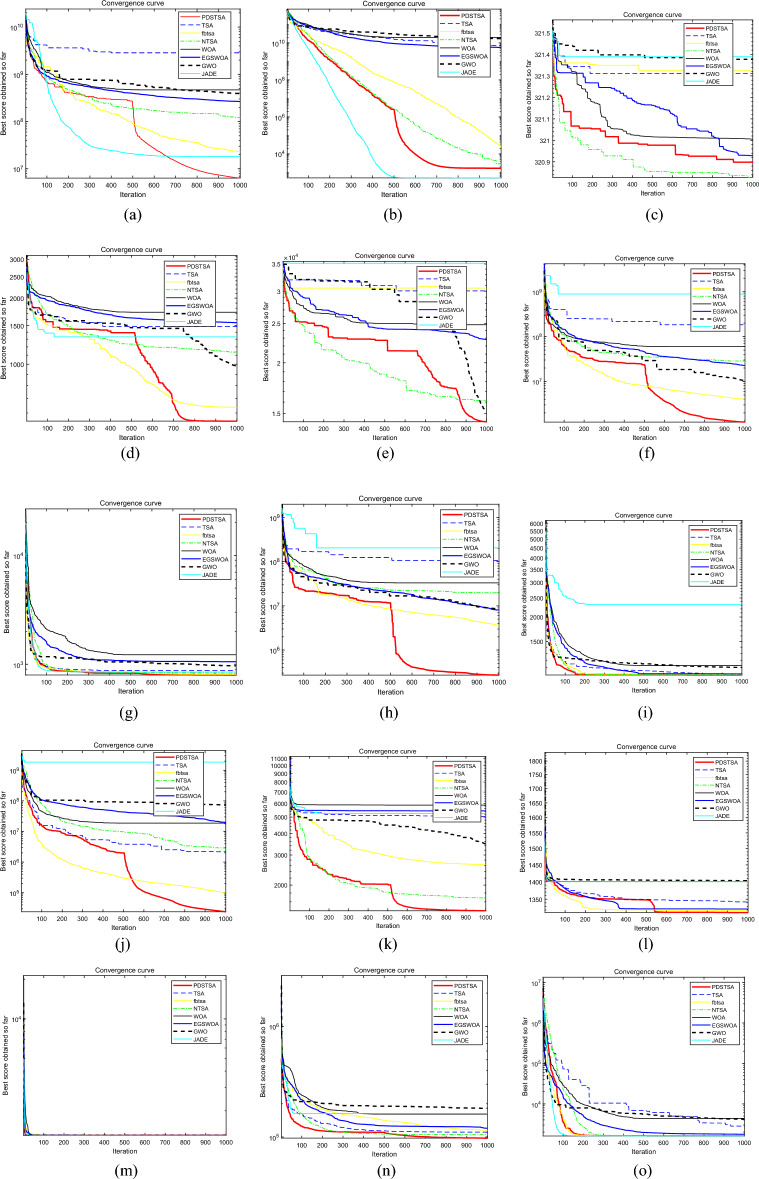
Comparison of convergence curves of various algorithms for solving CEC2015 test function. Figure shows the comparison of the convergence curves of PDSTSA, TSA, fbtsa, NTSA, WOA and EGSWOA algorithms for solving the CEC2015 test function when the dimension is 100, where Figure (**a**)–(**o**) correspond to the convergence curves of functions F1–F15 respectively.

The convergence curve intuitively reflects the algorithm’s ability to jump out of the local extreme value and the speed of downward convergence, which is an important indicator to measure the performance of the algorithm. The following is a comparison chart of convergence curves of eight algorithms for solving 15 functions in the CEC2015 suite when the dimension is 100, as shown in Fig. 1, and Fig. 1a–o corresponds to the convergence curve of functions F1–F15, respectively.

The convergence curves of the above 15 functions clearly show the variation trend of fitness values of PDSTSA, TSA, fbtsa, NTSA, WOA, and EGSWOA in the evolution process. It can be shown by the convergence curves of different functions that the convergence performance of the PDSTSA proposed in this paper is significantly better than other algorithms in most test functions.

In Fig. 1g,i,k,n, PDSTSA always has a faster convergence speed and higher optimization accuracy. TSA, WOA, EGSWOA, GWO and JADE converge slowly and quickly fall into local extreme values and cannot jump out, while fbtsa and NTSA converge slightly faster than TSA, WOA, EGSWOA, GWO and JADE. In Fig. 1b, the rate of convergence of PDSTSA is better than TSA, fbtsa, WOA, EGSWOA and GWO, and slightly inferior to JADE. In Fig. 1a,d, although the convergence speed of fbtsa, NTSA and JADE is faster than PDSTSA in the early stage of the iteration, in the middle and late stages of the iteration, PDSTSA can get rid of local extremum. The convergence speed and optimization accuracy are gradually better than fbtsa, NTSA and JADE, while TSA, WOA, EGSWOA and GWO fall into local optimization in the early stage of the iteration until the end of the iteration. In Fig. 1e, fbtsa, TSA, WOA, EGSWOA and JADE converge to local extreme value at the early stage of the iteration and cannot jump out. Although the convergence rate of NTSA is faster in the first 900 generations, the convergence rate is lower than PDSTSA when the iteration reaches 1000, and PDSTSA still has a downward convergence trend. Although GWO has a downward trend in the later stage, its optimization accuracy is not as good as PDSTSA within 1000 iterations. In Fig. 1f,h,j,l, the convergence speed of NTSA, TSA, WOA, EGSWOA, GWO and JADE is relatively slow. Although the convergence speed of fbtsa is faster than PDSTSA in the early stage of the iteration, PDSTSA can jump out of the local extreme value in the middle and late stages of the iteration, and the convergence speed and optimization precision are gradually better than fbtsa. In Fig. 1c,m,o, the convergence speed of PDSTSA is slightly slower than that of NTSA, but faster than the other six algorithms.

The above results and analysis show that the optimization accuracy and convergence speed of PDSTSA under high-dimensional conditions are significantly better than the other seven algorithms. This is mainly because the PDSTSA introduces the detection movement and pattern movement mechanism into the global search stage, which makes the individual search directional. At the same time, it reduces the step length $S$ in the optimization process, enhances the local search ability of the individual in the later stage of the iteration, and effectively improves the detection ability and convergence speed of the algorithm. The introduction of a random mutation strategy of individual dimension permutation increases the diversity of the population and improves the optimization performance of the algorithm. In the middle and later stages of the iteration, the elimination and update mechanism of inferior trees is used to obtain individuals with more search potential, enhance the ability of PDSTSA to jump out of the local extreme, and further improve search efficiency and convergence accuracy.

To sum up, PDSTSA has better optimization performance under both low-dimensional and high-dimensional conditions. Its solution accuracy, convergence speed, and optimization stability are better than those of TSA, NTSA, fbtsa, WOA, EGSWOA, GWO and JADE. The improvement mechanism of PDSTSA is obviously effective and superior.

### Wilcoxon rank sum test

Wilcoxon rank sum test^30^ can statistically analyze the significant difference between the two algorithms, which is a non-parametric test method. In this paper, the Wilcoxon rank sum test method is used to determine whether there is a significant difference between the experimental results of PDSTSA and the comparison algorithms. The data used in the rank sum test is the result of each algorithm running independently 30 times on the CEC2015 test function under 100-dimensional conditions. Table 3 shows the p-value of the rank sum test between PDSTSA and each comparison algorithm. When $p < 0.05$, the null hypothesis is rejected, there is a significant difference between the two algorithms. The symbols “+”, “=” and “−” after each data in the table indicate that the solution result of PDSTSA is superior to, equivalent to, and inferior to other corresponding algorithms in the table.

**Table 3:** Wilcoxon rank sum test results of PDSTSA and each algorithm at D = 100.

Function	TSA	fbtsa	NTSA	WOA	EGSWOA	GWO	JADE
F1	3.02E−11+	3.02E−11+	3.02E−11+	3.02E−11+	3.02E−11	1.95E−03	3.69E−11
F2	3.02E−11+	3.02E−11+	6.53E−07+	3.02E−11+	3.02E−11	1.53E−05	1.41E−09
F3	3.02E−11+	3.02E−11+	5.57E−10−	3.20E−09+	1.24E−03	7.39E−07	3.02E−11
F4	3.02E−11+	5.97E−09+	3.02E−11+	3.02E−11+	3.02E−11	2.01E−04	3.02E−11
F5	3.02E−11+	3.02E−11+	1.60E−07+	3.02E−11+	5.49E−11	3.02E−11	3.02E−11
F6	3.02E−11+	3.02E−11+	3.02E−11+	3.02E−11+	3.02E−11	3.02E−11	1.21E−10
F7	3.02E−11+	6.07E−11+	7.60E−07+	3.02E−11+	3.02E−11	2.62E−03	3.02E−11
F8	3.02E−11+	3.02E−11+	3.02E−11+	3.02E−11+	3.02E−11	3.02E−11	5.57E−10
F9	3.02E−11	3.02E−11+	3.02E−11+	3.02E−11+	3.02E−11	1.86E−01	3.02E−11
F10	3.02E−11+	7.09E−08+	3.02E−11+	3.02E−11+	3.02E−11	5.57E−10	4.57E−09
F11	3.02E−11+	3.02E−11+	3.02E−11+	3.02E−11+	3.02E−11	3.02E−11	3.02E−11
F12	3.05E−08+	1.07E−07−	9.06E−08+	5.49E−11+	3.02E−11	1.50E−07	2.40E−11
F13	3.02E−11+	3.02E−11+	3.02E−11−	3.02E−11+	3.02E−11	2.63E−11	2.52E−11
F14	3.02E−11+	3.02E−11+	8.15E−05+	3.02E−11+	3.02E−11	3.00E−11	2.96E−11
F15	3.02E−11+	4.50E−11−	3.02E−11+	3.02E−11+	3.02E−11	2.48E−07	3.15E−09

It can be seen from the statistical results in Table 3 that the *p*-value of PDSTSA and the other seven comparison algorithm are less than 0.05, and most of the symbols are “+”, indicating that PDSTSA has significant differences from other algorithms, and is obviously superior than other comparison algorithms.

### Applications in solving engineering optimization problems

In order to further verify the application ability and effectiveness of the proposed algorithm, the PDSTSA algorithm and the above seven comparison algorithms are used to solve engineering constrained optimization problems. When solving these engineering problems, the operation environment and parameter settings of each algorithm are the same as those in “Test function” section. Each algorithm runs independently 30 times to obtain the average value, the optimal value, and the worst value of its optimization design results. The design problems of alkylation unit operation and industrial refrigeration have complex design variables and constraints, which can well test the ability of the algorithm to solve the engineering constraint optimization problem.

#### Constraint treatment strategy

The mathematical model of engineering problems has many linear, nonlinear, equality, and inequality constraints. Using the evolutionary algorithm to solve constraint problems not only requires the performance of the algorithm but also depends on the constraint processing method. In this paper, the easy and effective outer point penalty function method is used to deal with the constraints in engineering problems and the constrained optimization problem is transformed into an unconstrained problem.

Firstly, the equality constraint is transformed into the following inequality constraint. The method of dealing with the equality constraint $h(x)$ is as follows:

10 $$\left| {h(x)} \right| - \delta \le 0$$

where $x$ is the candidate solution of the population, $\delta$ is the tolerance value of the equality constraint, generally taking a small positive number.

Then, the degree function $V_{i} (x)$ of the candidate solution $x$ in the population violating the $ith$ constraint is expressed as:

11 $$V_{i} (x) = \left\{ {\begin{array}{*{20}l} {\max \left\{ {0,g_{i} (x)} \right\}} \hfill & {1 \le i \le q} \hfill \\ {\max \left\{ {0,\left| {h_{i} (x)} \right| - \delta } \right\}} \hfill & {q + 1 \le i \le m} \hfill \\ \end{array} } \right.$$

where $q$ is the number of inequality constraints, and the number of equality constraints is $(m - q)$. If the candidate solution $x$ violates the constraint, there must be $V_{i} (x) > 0$. If the candidate solution doesn’t violate the constraint, then $V_{i} (x) = 0$. The penalty fitness function is constructed by introducing a penalty term into the objective function. The definition is as follows:

12 $$F(x) = f(x) + \sum\limits_{i = 1}^{m} {k \times V_{i} (x)}$$

where, $k$ is the penalty coefficient, and its value is a relatively large positive number. It can be found that the penalty value $\sum\nolimits_{i = 1}^{m} {k \times V_{i} (x)}$ is always a non-negative number. After replacement, the objective function value of the infeasible solution is greater than that of the feasible solution, effectively distinguish the feasible solution from the infeasible solution.

#### Solution and analysis of engineering constrained optimization problems

1. Optimal operation design of alkylation unit.

The optimized operation of the alkylation unit^31^ is widespread in the petroleum industry. The primary purpose of this problem is to increase the octane number of the olefin feedstock reacted with isobutene under acidic conditions. This problem is a complex optimization problem containing seven design variables and fourteen constraints. The design variables are olefin feed rate $x_{1}$, acid addition rate $x_{2}$, alkylate yield $x_{3}$, acid strength $x_{4}$, motor octane number $x_{5}$, external isobutene to olefin ratio $x_{6}$, F-4 performance number $x_{7}$. The mathematical model is defined as follows:

Design variables:

$$x = \left[ {x_{1} x_{2} x_{3} x_{4} x_{5} x_{6} x_{7} } \right]$$

Maximize:

$$f\left( x \right) = 0.035x_{1} x_{6} + 1.715x_{1} + 10.0x_{2} + 4.0565x_{3} - 0.063x_{3} x_{5}$$

Subject to:

$$g_{1} \left( x \right) = 0.0059553571x_{6}^{2} x_{1} + 0.88392857x_{3} - 0.1175625x_{6} x_{1} - x_{1} \le 0$$

$$g_{2} \left( x \right) = 1.1088x_{1} + 0.1303533x_{1} x_{6} - 0.0066033x_{1} x_{6}^{2} - x_{3} \le 0$$

$$g_{3} \left( x \right) = 6.66173269x_{6}^{2} - 56.596669x_{4} + 172.39878x_{5} - 10000 - 191.20592x_{6} \le 0$$

$$g_{4} \left( x \right) = 1.08702x_{6} - 0.03762x_{6}^{2} + 0.32175x_{4} + 56.85075 - x_{5} \le 0$$

$$g_{5} \left( x \right) = 0.006198x_{7} x_{4} x_{3} + 2462.3121x_{2} - 25.125634x_{2} x_{4} - x_{3} x_{4} \le 0$$

$$g_{6} \left( x \right) = 161.18996x_{3} x_{4} + 5000.0x_{2} x_{4} - 489510.0x_{2} - x_{3} x_{4} x_{7} \le 0$$

$$g_{7} \left( x \right) = 0.33x_{7} + 44.333333 - x_{5} \le 0$$

$$g_{8} \left( x \right) = 0.022556x_{5} - 1.0 - 0.007595x_{7} \le 0$$

$$g_{9} \left( x \right) = 0.00061x_{3} - 1.0 - 0.0005x_{1} \le 0$$

$$g_{10} \left( x \right) = 0.819672x_{1} - x_{3} + 0.819672 \le 0$$

$$g_{11} \left( x \right) = 24500.0x_{2} - 250.0x_{2} x_{4} - x_{3} x_{4} \le 0$$

$$g_{12} \left( x \right) = 1020.4082x_{4} x_{2} + 1.2244898x_{3} x_{4} - 100000x_{2} \le 0$$

$$g_{13} \left( x \right) = 6.25x_{1} x_{6} + 6.25x_{1} - 7.625x_{3} - 100000 \le 0$$

$$g_{14} \left( x \right) = 1.22x_{3} - x_{6} x_{1} + 1.0 \le 0$$

With bounds:

$$1000 \le x_{1} \le 2000,\quad 0 \le x_{2} \le 100,\quad 2000 \le x_{3} \le 4000,\quad 0 \le x_{4} \le 100$$

$$0 \le x_{5} \le 100,\quad 0 \le x_{6} \le 20,\quad 0 \le x_{7} \le 200$$

We first transform the maximization problem into a minimization problem to solve it. Table 4 shows the average, best, and worst values of the optimization results obtained by solving the optimization operation design problem of the alkylation unit after 30 runs of eight algorithms. From the data in the table, it can be seen that the average, optimal, and worst values obtained by PDSTSA are the theoretical optimal values and are superior to the other seven algorithms, indicating that the PDSTSA algorithm has excellent optimization ability and stability. The final design result of PDSTSA is the best and has a good effect on the optimization of the alkylation process.

**Table 4:** Optimization results of 8 algorithms for solving alkylation unit operation.

Algorithm	Average	Optimal	Worst
PDSTSA	− 4529.11974	− 4529.11974	− 4529.11974
TSA	− 4514.33982	− 4528.15011	− 4495.20182
fbtsa	− 68.89716	− 142.71933	263.72778
NTSA	− 4515.61273	− 4528.92699	− 4440.01832
WOA	1.75120E+18	− 4528.53100	5.25230E+18
EGSWOA	6.99850E+17	− 4520.82844	5.24870E+18
GWO	4.29814E+15	− 4528.18497	1.28476E+17
JADE	1.18716E+19	1.18306E+14	1.498197E+20

(B) Design of industrial refrigeration system.

Paul^32^ first described the mathematical model of the industrial refrigeration optimization design problem in 1987 and expressed the problem as a complex nonlinear inequality constrained optimization problem, including 14 design variables and 15 constraints. The specific mathematical model is as follows:

Design variable: $$x = [x_{1} x_{2} x_{3} x_{4} x_{5} x_{6} x_{7} x_{8} x_{9} x_{10} x_{11} x_{12} x_{13} x_{14} ]$$.

Minimize:

$$\begin{aligned} f(x) & = 63098.88x_{2} x_{4} x_{12} + 5441.5x_{2}^{2} x_{12} + 115055.5x_{2}^{1.664} x_{6} + 6172.27x_{2}^{2} x_{6} + 63098.88x_{1} x_{3} x_{11} \\ & \quad + 5441.5x_{1}^{2} x_{11} + 115055.5x_{1}^{1.664} x_{5} + 6172.27x_{1}^{2} x_{5} + 140.53x_{1} x_{11} + 281.29x_{3} x_{111} + 70.26x_{1}^{2} \\ & \quad {\kern 1pt} + 281.29x_{1} x_{3} + 281.29x_{3}^{2} + 14437x_{8}^{1.8812} x_{12}^{0.3424} x_{10} x_{14}^{ - 1} x_{1}^{2} x_{7} x_{9}^{ - 1} + 20470.2x_{7}^{2.893} x_{11}^{0.316} x_{1}^{2} \\ \end{aligned}$$

Subject to:

$$g_{1} (x) = 1.524x_{7}^{ - 1} \le 1,$$

$$g_{2} (x) = 1.524x_{8}^{ - 1} \le 1,$$

$$g_{3} (x) = 0.07789x_{1} - 2x_{7}^{ - 1} x_{9} - 1 \le 0,$$

$$g_{4} (x) = 7.05305x_{9}^{ - 1} x_{1}^{2} x_{10} x_{8}^{ - 1} x_{2}^{ - 1} x_{14}^{ - 1} - 1 \le 0,$$

$$g_{5} (x) = 0.0833x_{13}^{ - 1} x_{14} - 1 \le 0,$$

$$g_{6} (x) = 47.136x_{2}^{0.333} x_{10}^{ - 1} x_{12} - 1.333x_{8} x_{13}^{2.1195} + 62.08x_{13}^{2.1195} x_{12}^{ - 1} x_{8}^{0.2} x_{10}^{ - 1} - 1 \le 0,$$

$$g_{7} (x) = 0.04771x_{10} x_{8}^{1.8812} x_{12}^{0.3424} - 1 \le 0,$$

$$g_{8} (x) = 0.0488x_{9} x_{7}^{1.893} x_{11}^{0.316} - 1 \le 0,$$

$$g_{9} (x) = 0.0099x_{1} x_{3}^{ - 1} - 1 \le 0,$$

$$g_{10} (x) = 0.0193x_{2} x_{4}^{ - 1} - 1 \le 0,$$

$$g_{11} (x) = 0.0298x_{1} x_{5}^{ - 1} - 1 \le 0,$$

$$g_{12} (x) = 0.056x_{2} x_{6}^{ - 1} - 1 \le 0,$$

$$g_{13} (x) = 2x_{9}^{ - 1} - 1 \le 0,$$

$$g_{14} (x) = 2x_{10}^{ - 1} - 1 \le 0,$$

$$g_{15} (x) = x_{12} x_{11}^{ - 1} - 1 \le 0$$

With bounds:

$$0.001 \le x_{i} \le 5,i = 1,2,\ldots,14$$

Table 5 shows the average, best, and worst values of the optimization results obtained by the eight algorithms that have been run independently 30 times to solve the optimization design problem of the industrial refrigeration system. It can be seen from the data in the table that, except for the relatively poor WOA solution results, the final results obtained by other algorithms are relatively similar, but the average value, the optimal value, and the worst value of PDSTSA are all theoretical optimal values. In contrast, other algorithms cannot achieve the theoretical optimal value, indicating that the algorithm in this paper performs better in solving the constrained optimization problems of complex engineering design.

**Table 5:** Optimization results of 8 algorithms for industrial refrigeration system design.

Algorithm	Average	Optimal	Worst
PDSTSA	0.032213000	0.032213000	0.032213000
TSA	0.032274189	0.032213012	0.033099035
fbtsa	0.033121707	0.032716582	0.033566852
NTSA	0.034014452	0.032213001	0.044092606
WOA	2.81026E+14	0.036733519	9.38669E+14
EGSWOA	0.034985849	0.032230973	0.044783916
GWO	2.18447E+14	0.034391062	9.36228E+14
JADE	4.76918E+13	0.208500543	1.43075E+15

## Conclusion

In order to improve the shortcomings of TSA, which is easy to fall into local optimum and slow convergence speed, this paper proposes the PDSTSA algorithm. In terms of algorithm improvement, firstly, a pattern search strategy is introduced in the global search stage, and the population explores along the direction of making the fitness value tend to the optimal value, improving the algorithm’s convergence speed. Then, individuals undergo random mutation in a round of iterations, increasing the diversity of population positions. Finally, the individuals that still don’t have the search ability in the middle and later stages of the iteration are eliminated, and then the new individuals with search potential are supplemented to improve the quality of the solution and the ability of the algorithm to jump out of the local extreme. In terms of global optimization, PDSTSA and seven other comparison algorithms with superior performance are simulated on the IEEE CEC2015 test function set suite. From the optimization accuracy analysis and convergence curve comparison of the experimental results, it can be seen that PDSTSA significantly improved optimization accuracy and convergence speed. Then, the Wilcoxon rank sum test proves a significant difference between the optimization results of PDSTSA and the comparison algorithms. Finally, in engineering design applications, the advantages and potential of PDSTSA are further demonstrated by solving engineering design problems. In the follow-up work, we will conduct more in-depth research on TSA, continue to improve and perfect the algorithm in this paper, and apply it to more practical optimization problems reasonably to improve its optimization performance and expand its application fields continuously.

## Data Availability

The datasets generated during and analyzed during the current study are available from the corresponding author on reasonable request.

## References

[1] Kirkpatrick S, Gelatt CD, Vecchi MP (1983). Optimization by simulated annealing. Science.

[2] Kennedy, J. & Eberhart, R. Particle swarm optimization. In *Icnn95-International Conference on Neural Networks* (IEEE, 1995)

[3] Yang X-S (2011). Bat algorithm for multi-objective optimisation. Int. J. Bio-Inspired Comput..

[4] Yang, X.-S. Flower pollination algorithm for global optimization. In *Unconventional Computation and Natural Computation: 11th International Conference* (Springer, 2012).

[5] Mirjalili S, Lewis A (2016). The whale optimization algorithm. Adv. Eng. Softw..

[6] Mirjalili S (2017). Salp swarm algorithm: A bio-inspired optimizer for engineering design problems. Adv. Eng. Softw..

[7] Kiran MS (2015). TSA: Tree–seed algorithm for continuous optimization. Expert Syst. Appl..

[8] Chen X (2022). Forecasting short-term electric load using extreme learning machine with improved tree seed algorithm based on Lévy flight. Eksploat. Niezawodn..

[9] Koc I, Atay Y, Babaoglu I (2022). Discrete tree seed algorithm for urban land readjustment. Eng. Appl. Artif. Intell..

[10] Lenin K (2021). Real power loss reduction by hybridization of tree–seed algorithm with sine–cosine algorithm. J. Electr. Power Energy Syst..

[11] Zhou J, Zheng Y, Xu Y, Liu H, Chen D (2018). A heuristic TS fuzzy model for the pumped-storage generator-motor using variable-length tree–seed algorithm-based competitive agglomeration. Energies.

[12] Muneeswaran, V. & Pallikonda Rajasekaran, M. Beltrami-regularized denoising filter based on tree seed optimization algorithm: An ultrasound image application. In *Information and Communication Technology for Intelligent Systems* (Springer, 2018).

[13] El-Fergany AA, Hasanien HM (2018). Tree–seed algorithm for solving optimal power flow problem in large-scale power systems incorporating validations and comparisons. Appl. Soft. Comput..

[14] Gharehchopogh FS (2022). Advances in tree seed algorithm: A comprehensive survey. Arch. Comput. Method Eng..

[15] Jiang J, Meng X, Qian L, Wang H (2022). Enhance tree–seed algorithm using hierarchy mechanism for constrained optimization problems. Expert Syst. Appl..

[16] Beşkirli A, Özdemir D, Temurtaş H (2020). A comparison of modified tree–seed algorithm for high-dimensional numerical functions. Neural Comput. Appl..

[17] Babalik A, Cinar AC, Kiran MS (2018). A modification of tree–seed algorithm using Deb’s rules for constrained optimization. Appl. Soft. Comput..

[18] Jiang J (2020). Enhancing tree–seed algorithm via feed-back mechanism for optimizing continuous problems. Appl. Soft. Comput..

[19] Kiran MS, Hakli H (2021). A tree–seed algorithm based on intelligent search mechanisms for continuous optimization. Appl. Soft. Comput..

[20] Ding Z, Li J, Hao H, Lu ZR (2019). Nonlinear hysteretic parameter identification using an improved tree–seed algorithm. Swarm Evol. Comput..

[21] Horng SC, Lin SS (2018). Embedding ordinal optimization into tree–seed algorithm for solving the probabilistic constrained simulation optimization problems. Appl. Sci..

[22] Cinar AC, Kiran MS (2018). Similarity and logic gate-based tree–seed algorithms for binary optimization. Comput Ind Eng..

[23] Gungor I, Emiroglu BG, Cinar AC, Kiran MS (2020). Integration search strategies in tree seed algorithm for high dimensional function optimization. Int. J. Mach. Learn. Cybern..

[24] Hooke R, Jeeves TA (1961). “Direct Search” solution of numerical and statistical problems. J. ACM..

[25] Zhang DL, Xia HW, Zhang CX, Ma GC, Wang CH (2022). Improved firefly algorithm and its convergence analysis. J. Syst. Eng. Electron..

[26] Liang, J. J., Qu, B. Y., Suganthan, P. N. & Chen, Q. Problem definitions and evaluation criteria for the CEC 2015 competition on learning-based real-parameter single objective optimization. *Technical Report201411A, Computational Intelligence Laboratory, Zhengzhou University, Zhengzhou China and Technical Report, Nanyang Technological University, Singapore*, Vol. 29, 625–640 (2014).

[27] Xiao ZY, Liu S (2019). Study on elite opposition-based golden-sine whale optimization algorithm and its application of project optimization. Acta Polym. Sin..

[28] Mirjalili S, Mirjalili SM, Lewis A (2014). Grey wolf optimizer. Adv. Eng. Softw.

[29] Zhang J, Sanderson AC (2009). JADE: adaptive differential evolution with optional external archive. IEEE Trans. Evolut. Comput..

[30] Derrac J, García S, Molina D, Herrera F (2011). A practical tutorial on the use of nonparametric statistical tests as a methodology for comparing evolutionary and swarm intelligence algorithms. Swarm Evol. Comput..

[31] Babu BV, Angira R (2006). Modified differential evolution (MDE) for optimization of non-linear chemical processes. Comput. Chem. Eng..

[32] Pandrei N, Andrei N (2013). Nonlinear Optimization Applications Using the GAMS Technology.

